# 

**Published:** 1890-03

**Authors:** 


### CONTENTS.

### *EDITORIALS.*

PAGE

Argument and Reasoning, . . . . . . . . . . . 97
The Difference Between the Old School and the New, . . . . 100
The Unattainable, . . . . . . . . . . . . 101
The Minuteness of the Dose, . . . . . . . . . . 102
Quinine, . . . . . . . . . . . . . . 102
La Grippe, . . . . . . . . . . . . . . 102
Intermittent Fever Treated by Friction, . . . . . . . 103
The Definition of a Homœopathic Physician, . . . . . . 103

### *MISCELLANEOUS.*

Of the Drug Curative. Dr. P. P. Wells, communicated by C. Carleton Smith, M. D., . . . . . . . . . . . 104
Some Relations of Hysteria. J. M. Dutton, M. D., . . . . . 108
Notes from Past Meetings of The Lippe Society, . . . . . 115
How to Select the Proper Potency, . . . . . . . . . 119
La Grippe. Samuel Swan, M. D., . . . . . . . . . 124
La Grippe. H. B. Stiles, M. D., . . . . . . . . . 127
La Grippe, . . . . . . . . . . . . . . 128
Flatulent Dyspepsia. Dr. Jose Nadal, . . . . . . . . 128
Aconite in Its Relation to the Throat. E. J. L., . . . . . 131
What is the Simillimum? M. R. R. Wolff, M. D., . . . . . 132
Clinical Cases. G. W. Sherbino, M. D., . . . . . . . . 133
An Unusual Symptom. Edward T. Balch, M. D., . . . . . 136
Proving of Saccharum Lactis. B. Fincke, M. D., . . . . . 137
In Memoriam—L. S. Reynolds, M. D., . . . . . . . . 139
International Hahnemannian Association. T. Dwight Stowe, M. D., 139
Query for the Readers of The Homœopathic Physician. John Hall, Sr., M. D., . . . . . . . . . . . . 140

### *BOOK NOTICES.*

El Consultor Homœopatico, . . . . . . . . . . 141
The Annals of Surgery, . . . . . . . . . . . 141
Harper's Magazine for February, . . . . . . . . . 141
The National Magazine for January, . . . . . . . . 142
Revue Homœopathique Francaise, . . . . . . . . 142
Disease and Sickness. By Samuel Swan, M. D., . . . . . . 142
The Memphis Journal of the Medical Sciences, for February, . . 142

### *NOTES AND NOTICES.*

The Psychology of Epidemics—Dr. W. B. Clarke—A Snapping Turtle Baby—M. Pasteur—Treatment of Stammering, . . . . 143–144

